# Relationship between virtual reality balloon analogue risk task and risky decision-making

**DOI:** 10.1371/journal.pone.0282097

**Published:** 2023-02-22

**Authors:** Uijong Ju, Christian Wallraven

**Affiliations:** 1 Department of Information Display, Kyung Hee University, Seoul, South Korea; 2 Department of Brain and Cognitive Engineering, Korea University, Seoul, South Korea; 3 Department of Artificial Intelligence, Korea University, Seoul, South Korea; Qatar University, QATAR

## Abstract

The balloon analogue risk task (BART) is widely used to assess risk-taking tendencies on behavioral tests. However, biases or unstable results are sometimes reported, and there are concerns about whether the BART can predict risk behavior in the real world. To address this problem, the present study developed a virtual reality (VR) BART to enhance the reality of the task and narrow the gap between performance on the BART and risk behavior in the real world. We evaluated the usability of our VR BART through assessments of the relationships between BART scores and psychological metrics and additionally implemented an emergency decision-making VR driving task to investigate further whether the VR BART can predict risk-related decision-making in emergency situations. Notably, we found that the BART score significantly correlated with both sensation-seeking and risky driving behavior. Additionally, when we split participants into groups with high and low BART scores and compared their psychological metrics, we found that the high-score BART group included more male participants and exhibited higher sensation-seeking and more risky decision-making in an emergency situation. Overall, our study shows the potential of our new VR BART paradigm to predict risky decision-making in the real world.

## Introduction

Predicting risk-taking behavior in the real world is important to prevent problems caused by risky behavior. While the easiest way to measure risk-taking tendencies is the use of self-report questionnaires, there is an ongoing debate about the reliability of such self-reports [1], and a previous study showed that across the lifespan, there is only a small correlation between self-reported risk-taking and other measurements of risk-taking behavior [2]. Therefore, it is questionable whether self-reported risk-taking can predict actual risk-taking behavior in the real world, and several behavioral tests have been developed to predict risky behavior in real-world situations more accurately.

The balloon analogue risk task (BART) [3] is a widely used test to assess risk-taking tendencies and predict risky behavior in real-world situations. During the BART, participants must inflate a balloon to receive a reward for each pump but lose their accumulated gains from a trial if the balloon pops before they stop pumping. Participants, therefore, must balance between taking a risk for a higher reward and preventing a loss. The BART is used in a variety of fields to predict risk-taking behavior when it comes to alcohol consumption [4], gambling [5], addiction [6], smoking [7], and sexual behaviors [8]; however, compared to the original study [3], later research reported relatively small effect sizes for the BART: for example, in a study on risk-taking related to personality traits, sensation seeking and impulsivity showed only moderate correlations on the original BART [3]. Similarly, in a meta-analysis, the BART showed relatively small effect sizes for sensation seeking and impulsivity [9] overall. Since sensation seeking and impulsivity are personality traits that are correlated with risky driving as well as with accident outcomes [10, 11], drug use in aggressors [12], alcohol use, and related risky behaviors [13, 14], these results imply that predictions of risk-taking behavior in real-world situations based on the original BART may be biased and inconsistent across studies [15]. To solve this problem, several studies employed modified versions of the BART that change the inflation process of the balloon from manual to automatic [15], provide participants with explicit information about explosion probabilities [16], or decrease the total possible number of pumps [17, 18]. While these studies report less biased results, whether the modified BART can accurately predict decision-making in the real world remains questionable. One novel approach to increasing the reliability of the BART would be to enhance the participant’s emotional engagement with the task—an approach that has been suggested for bridging the gap between models and real-world risk-taking behaviors [19]. Previous meta-analysis studies conclude that reliable associations between personality traits and BART scores are limited to studies that elicit higher emotional arousal [9], implying that higher emotional arousal may enhance the validity of the test. While the best way to elicit such increased emotional arousal would be to test participants on the BART in the real world, the balloon explosions can potentially shock participants. Moreover, it is difficult to make balloons explode randomly, which poses another challenge for real-world applications of the BART.

One possible solution to solve this problem is using virtual reality (VR) to implement the BART in a realistic environment without causing any actual harm. Using a VR setup makes it possible to expand the balloon towards the participant, which can induce real anxiety or fear about a potential explosion, and realistic pump and explosion sounds can be provided in immersive virtual environments to maximize tension compared to a typical computer desktop BART. Therefore, in the current study, we developed a VR BART and investigated associations between personality traits and adjusted pump numbers on the task to evaluate the validity of our VR BART to assess risk-taking. We hypothesized that VR BART scores are associated with the same personality traits as in the original study [3], including sensation-seeking and impulsivity.

Another advantage of VR over questionnaires is that VR tasks allow for the investigation of actual risky decision-making in unpredictable situations, which we did here to investigate the relationship between BART scores and risk-taking behaviors in VR. To implement risky naturalistic decision-making that comes close to real-world situations, we chose a VR driving simulation, showing ecological validity in reflecting real-world driving [20]. We further hypothesized that an association between BART scores and the outcomes of risky decision-making can be found in VR driving.

Additionally, since the original BART showed biases in pump scores [15], which make it difficult to find correlations with risky behavior, we divided our participant sample into a “high-BART” and a “low-BART” group to investigate overall differences in psychological metrics and risk behavior. Psychological metrics included sensation seeking [11, 21–24] and impulsivity [10, 25–27] personality traits as well as age [28–30] and sex [28, 31–33], all of which have already shown to be associated with general risky behavior, and in particular, risky driving in the real world. We hypothesized that the high- and low-BART subgroups differ in psychological metrics and risky decision-making.

Overall, three hypotheses were posed for the present study. First, there is a relationship between personality traits and VR BART scores. Second, risky decision-making in VR is associated with BART scores. Third, groups with high and low BART scores differ in psychological metrics and risky decision-making behavior. In summary, our study aimed to develop a new VR version of the BART to assess risk-taking tendencies and predict risky behavior in real-world situations.

## Materials and methods

### Experimental design

**Fig 1:**
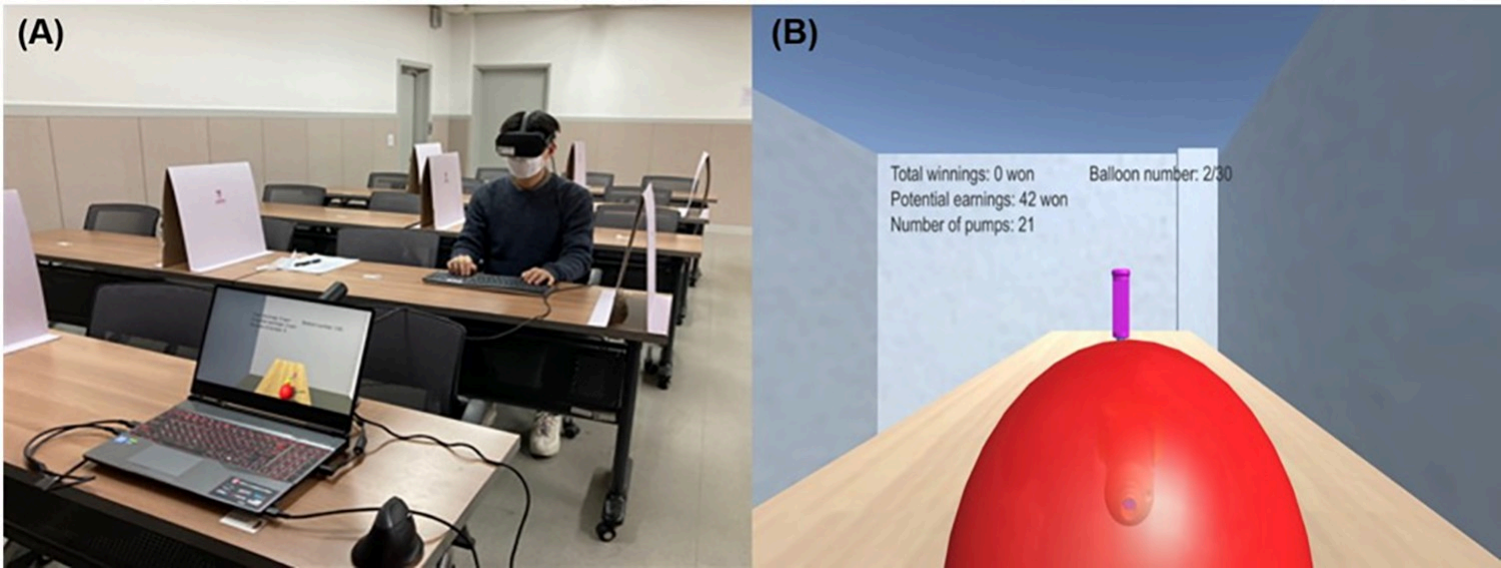
Setup of the experiment. (A) Setup of a VR BART (B) Screenshot of a VR BART trial.

We used a 3D environment development tool, Unity3D 2018.2.0.f3 (Unity Technologies, San Francisco, USA) to create our VR BART. To implement an environment in VR that mimics a real-world laboratory, we put a balloon pump on the table in front of the participants that had a visual representation that was very similar to the actual experiment table (see Fig 1). Subsequently, we used 3D balloons that are freely available on the Unity platform and the balloon pump provided on GrabCAD website (https://grabcad.com/library/ultimaker-balloon-pump-1) to implement the balloon inflation as a result of the pumping action. Later, to enhance the immersion and reality of the task, when participants pressed the “pump” button, we enlarged the balloon towards them and added realistic inflation (https://www.youtube.com/watch?v=i5DhmxAVC20) and popping (https://www.youtube.com/watch?v=YzpwI25UmoE) sounds, both extracted from YouTube videos. We added sound to the 3D scene and played this through the VR headset earphones to enhance tension. Participants wore an Oculus Rift (Oculus Rift CK1; Irvine, USA) headset and viewed the scene at 1080x1200 pixel resolution with a frame rate of 90 Hz. Head movements were translated into camera movements in the virtual environment, further increasing immersion. Our BART consisted of 30 trials of balloon pumping, with balloon popping occurring randomly after 1–128 pumps, and each pump added 2 points to the participant’s accumulated rewards for the current trial. Additionally, we displayed the current trial number, the current number of pumps, and the potential and total rewards to increase the participants’ motivation. Participants used the “space” key on the keyboard to inflate the balloon and the “enter” key to stop pumping and move to the subsequent trial (see Fig 1). The experiment ended automatically after 30 trials.

### Sample size and participants

A correlation bivariate normal model in G*power was used to calculate the required sample size based on the potential correlation between personality scales and BART scores. Following a meta-analysis that showed positive correlations between sensation seeking, impulsivity, and BART scores and reported effect sizes from 0.11 to 0.31 for a mean age above 20 for sensation seeking and from 0.01 to 0.39 for a mean age above 20 for impulsivity, and consequently set the effect size to 0.2 and the power to 80% one-tailed [9]. The estimated required sample size for the current study was 153, and we recruited 170 participants (115 males and 55 females; mean age = 22.7; SD = 2.66). In particular, age was limited to young adults since a previous study showed a higher effect size of the correlation between BART and sensation seeking and impulsivity only for older adolescents and young adults [9] and that young adults have higher affinity towards risky driving [34–36].

### Psychological metrics

Based on the original BART study [3], we chose the personality factors sensation seeking [37] and impulsivity [38], which are potentially associated with BART scores and risky decision-making. To accurately measure these personality traits, we used the validated Korean versions of the sensation-seeking scales translated from Zuckerman’s sensation-seeking scale Ⅴ [37], which had a Cronbach’s alpha between 0.67–0.83 [39], and impulsivity scales translated from Barratt Impulsiveness Scale-11 [38], validated for a Cronbach’s alpha between 0.58–0.80 and test-retest reliability 0.89–0.95 [40]. Additionally, a literature review showed that age [28–30] and sex [28, 31–33] might influence risk-taking tendencies; therefore, the potential relationships of age and sex with BART scores were also investigated.

### Risk-taking measurements

**Fig 2:**
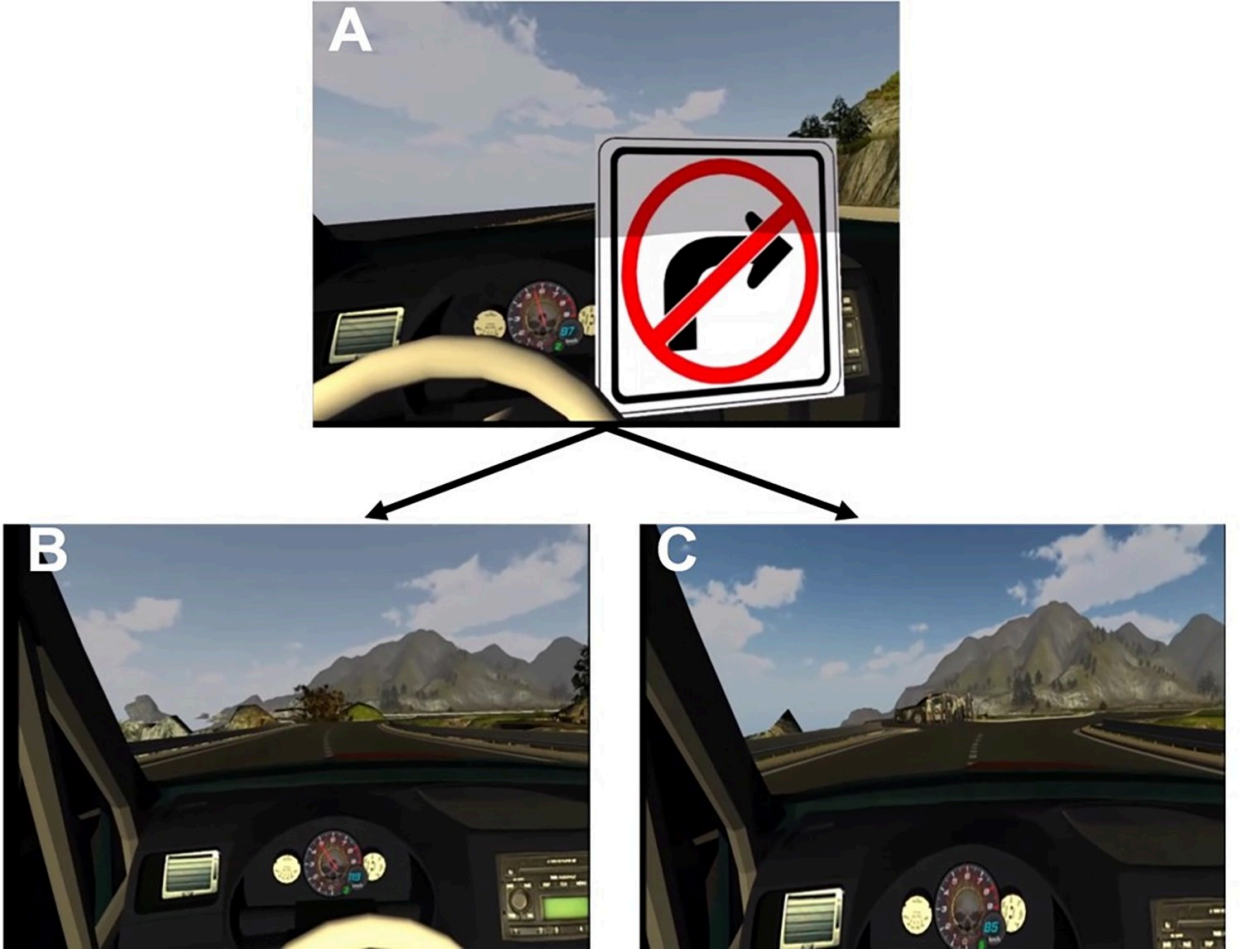
Visual display during the emergency situation at the end of the test session. (A) Warning signal before the event. (B) Trees blocking the left road. (C) Pedestrians blocking the left road.

Subsequently, risk-taking behavior during VR driving was investigated to scrutinize the relationship between risk-taking behavior and BART scores. The VR driving task was performed several days after the BART to minimize potential task influences. During the VR driving task, before the test session, participants first underwent three training trials to learn to drive in VR. Participants were instructed before entering the training session that the goal of the training was to learn how to drive in VR and informed that several randomly determined forks would appear on the road and that if they entered the wrong road at a fork, their vehicle would fall off the cliff on which they were driving. Participants were also informed that they needed to complete the entire course in at least one trial without failing to finish the training session. Additionally, to prevent participants from falling off the cliff, visual and auditory warning signs were presented before each fork appeared to indicate which direction participants had to choose to stay safely on the cliff (see Fig 2A). After finishing the training session, participants went through a test session, where they were informed that their lap time would be recorded to study their driving behavior. Notably, to induce realistic risky decision-making, one experiment’s goal was hidden from participants, who were unexpectedly presented with an accident situation. During the test session, at the final fork, pedestrians or trees suddenly blocked the road leading away from the end of the cliff (i.e., the “safe” road; see Fig 2B and 2C). Participants could hit the brake, but the brake sensitivity was low, and since the road leading to the fork was straight, they entered the emergency situation at a fast speed, making it impossible to stop the car before deciding which road to choose. Participants could either choose the road that would lead them to the end of the cliff and try to stop the vehicle or choose the safe direction away from the end of the cliff and try to stop the car before the collision. After the participant chose a direction, the experiment automatically finished before the actual collision or fall from the cliff happened. We recorded a video of each participant’s driving behavior to classify their decision-making and assigned participants who chose the road leading to the end of the cliff to the high-risk-taking group based on risk evaluation of a similar event situation in our previous study [41]. Additionally, we recorded the participants’ average driving speed in the emergency situation, starting from the sound and visual warning to the end of the trial, to further investigate the relationship between driving speed in risky situations and BART scores, as explored in our previous study on decision-making during driving that forced participants to choose between sacrificing self or pedestrians [42].

### Procedure

Before entering the study, participants were informed about the aims of the experiment and provided written consent. They were told that the aim of the BART was to collect as many points as possible, that they could stop pumping to collect their accumulated rewards for each trial before the balloon exploded, and that if a balloon exploded, the rewards for the current trial were lost. After participants confirmed that they understood the task and before the actual BART, they underwent a short training session. After the training, participants performed the BART, followed by a personality questionnaire potentially related to BART scores. Subsequently, two or three days after the BART, participants revisited our laboratory and performed the VR driving task. During the driving task, participants were informed about the aim of the study and learned how to drive in VR. They then completed three training trials to familiarize themselves with driving in VR. After each training trial, the experimenter asked them how they felt and gave them extra time to rest when they reported experiencing 3D sickness. After participants finished the training session, they entered the test session without being informed about the emergency situation. After the test session, the experimenter checked on the participants once more to minimize potential emotional impact; none of the participants reported any problems [42].

### Ethical statement

This study was approved by the local ethics committee of Korea University (approval number: KU-IRB-2018-0096-02). All methods and procedures were carried out in accordance with the relevant guidelines and regulations.

### Data analysis

Age, gender, risky decision-making during emergency situations, driving speed during emergency situations, sensation-seeking, and impulsivity were analyzed to investigate the relationship between BART scores and psychological metrics. Prior to the statistical analysis, Shapiro–Wilk tests were conducted to confirm normality and decide on types of correlational analysis. Spearman correlations were used for analyses of the relationship between BART scores and psychological metrics using SPSS (Version 26.0, SPSS Inc., Chicago, IL, USA). Subsequently, a multiple linear regression analysis was conducted to confirm that BART scores can significantly predict psychological metrics. Further, median value was utilized to split participants into high- and low-BART score groups typically used for group split [43] and independent sample *t*-tests were conducted to analyze the differences between the high- and low-score groups. Finally, a step-wise backward logistic regression analysis was conducted to analyze whether risky emergency decision-making can be predicted using psychological metric data and the division of high- and low-BART score groups.

## Results

### Descriptive analysis

The descriptive statistical data for all 170 participants are listed in Table 1.

**Table 1:** Descriptive statistics of all participants.

Total number	170
Sex (male/female)	115/55
Age (std)	22.70 (2.66)
BART (std)	27.00 (11.82)
Risky decision-making during emergency—yes/no	66/104
Driving speed during emergency (test)–km/h (std)	91.49 (28.18)
Sensation seeking (std)	52.69 (12.42)
Impulsivity (std)	35.66 (10.90)

Personality scales are rescaled to 1–100% for easier comparison; BART, balloon analogue risk task

First, the normality of the BART score data was checked to determine the required type of correlational analysis; the Shapiro–Wilk test (*p* < .001) confirmed that the BART scores were not normally distributed. Second, to investigate the relationship between the BART scores and the psychological metrics, Spearman correlations were conducted between the two (see Table 2), which are adequate for non-normally distributed data. It was found that sensation seeking (r = 0.16, *p* = .034), driving speed during the emergency (r = 0.15, *p* = .048), and risky decision-making (choice of the road during the emergency) significantly correlated with the BART scores (r = 0.15, *p* = .049).

**Table 2:** Correlations between driving experience, personality traits, age, sex, BART scores, and risky decision-making.

	B_1_	X_1_	X_2_	P_1_	P_2_	R_1_
B_1_ BART scores						
X_1_ Sensation seeking	0.16*					
X_2_ Impulsivity	0.02	0.22**				
P_1_ Age	-0.01	0.22**	-0.02			
P_2_ Sex	-0.14	-0.19**	-0.01	-0.25**		
R_1_ Risky decision in the event	0.15*	0.04	-0.08	0.01	0.06	
R_2_ Driving speed in risky situation	0.15*	0.15	0.06	-0.08	-0.10	0.06

* p < 0.05, ** p < 0.01; BART, balloon analogue risk task

### Prediction of BART scores from psychological metrics

A multiple linear regression analysis was run to examine whether psychological metrics can predict BART scores (see Table 3). The overall regression model showed trends towards correct predictions of BART scores [R^2^ = 0.066, F(6,163) = 1.918, *p* = .081], and only sensation-seeking significantly predicted the scores (β = .20, *p* = .017).

**Table 3:** Regression analysis for predicting BART scores from psychological metrics.

	BART score			
	Regression coefficient β	Statistical significance p	Low CI	High CI
Sensation-seeking	0.173	.037*	.025	.786
Impulsivity	-0.034	.669	-.229	.147
Sex	-0.070	.381	-5.747	2.207
Age	-0.103	.195	-1.154	.237
Emergency driving speed	0.084	.276	-1.637	5.697
Risky decision in the event	0.102	.194	-.022	.107

* p < 0.05; BART, balloon analogue risk task; Upper and lower 95% confidence intervals (CI) are displayed in the last two columns.

### Differences in psychological metrics between the high-and low-BART subgroups

Subsequently, the participants were divided into the high- and low-BART subgroups and psychological metrics were compared to assess potential differences (see Table 4). Chi-square tests showed significant group differences for risky decision-making (*χ2* = 5.408, *p* = .027) and sex (*χ2* = 5.538, *p* = .022): the high-BART group exhibited more risky decision-making and contained significantly more male participants. Then, independent sample *t*-tests were run to investigate whether the high- and low-BART groups exhibited different personality features and driving speeds during the emergency situation. It was found that the high-BART group had significantly higher sensation-seeking scores than the low-BART group (*t*[168] = 2.341, *p* = .020), whereas impulsivity (*t*[168] = 0.361, *p* = .718), age (*t*[168] = 1.379, *p* = .170), and driving speed in the emergency situation (*t*[168] = 1.542, *p* = .125) showed no significant differences.

**Table 4:** Descriptive statistics for the high- and low-BART groups.

	High-BART	Low-BART
Total number	84	86
BART score (std)	**36.39 (5.06)**	**17.84 (9.06)**
Age (std)	22.42 (1.90)	22.98 (3.21)
Sex (male/female)	**64/20**	**51/35**
Risky decision-making in emergency—yes/no	**40/44**	**26/60**
Average driving speed (std)	65.45 (9.02)	62.73 (10.80)
Emergency driving speed (std)	94.85 (30.67)	88.21 (25.26)
Sensation seeking (std)	21.87 (9.32)*	**20.08 (8.95)**
Impulsivity (std)	61.83 (10.78)	62.38 (8.98)

*Significant differences between the two groups are indicated in bold fonts.

### Prediction of risky decision-making from psychological metrics

As the previous analysis showed that BART scores could be partly predicted from psychological metrics, next it was investigated whether it was possible to predict the participants’ risky decision-making during the emergency situation from the psychological metrics. Stepwise backward logistic regression was used for this analysis, eliminating the non-significant values to find regression models. Results showed that only BART group assignment showed significance across all steps (Model 1: *p* = .019; Model 2: *p* = .014; Model 3: *p* = .017; Model 4: *p* = .012; Model 5: *p* = .012; Model 6: *p* = .021) and only Model 5 showed statistically significant (*χ2* = 7.287, *p* = .026) and good model fit (Hosmer–Lemeshow goodness-of-fit *p* = 0.07). The analysis results indicate that the BART subgroup assignment with sex significantly predicted risky decision-making (see Table 5), which confirms our earlier findings.

**Table 5:** Logistic regression results for predicting risky decision-making in the emergency situation from psychological metrics.

Models	Psychological metrics	*β*	*p*-value	OR (95% CI)
Model 1	Sensation seeking	0.02	.614	1.02 (0.95–1.09)
	Impulsivity	-0.03	.162	0.98 (0.94–1.01)
	Sex	-0.63	.092	0.54 (0.26–1.11)
	Age	0.04	.514	1.05 (0.92–1.19)
	Emergency driving speed	0.01	.283	1.01 (1.00–1.02)
	BART group assignment	0.81	.019*	2.24 (1.14–4.40)
	Constant	-0.86	.650	
Model 2	Impulsivity	-0.02	.190	0.98 (0.95–1.01)
	Sex	-0.60	.103	0.55 (0.27–1.13)
	Age	0.05	.443	1.05 (0.93–1.19)
	Emergency driving speed	0.01	.253	1.01 (1.00–1.02)
	BART group assignment	0.84	.014*	2.31 (1.19–4.49)
	Constant	-0.84	.656	
Model 3	Impulsivity	-0.02	.194	0.98 (0.95–1.01)
	Sex	-0.53	.135	0.59 (0.29–1.18)
	Emergency driving speed	0.01	.286	1.01 (0.99–1.02)
	BART group assignment	0.80	.017*	2.22 (1.15–4.28)
	Constant	0.28	.811	
Model 4	Impulsivity	-0.02	.220	0.98 (0.95–1.01)
	Sex	-0.50	.153	0.61 (0.30–1.20)
	BART group assignment	0.83	.012*	2.30 (1.20–4.41)
	Constant	0.74	.500	
Model 5	Sex	-0.47	.175	0.62 (0.31–1.24)
	BART group assignment	0.83	.012*	2.30 (1.20–4.39)
	Constant	-0.57	.063	
Model 6	BART group assignment	0.74	.021*	2.10 (1.12–3.93)
	Constant	-0.84	.000*	

p < 0.05, OR,odds ratio; CI, confidence interval; BART, balloon analogue risk task

## Discussion

This study developed a new VR BART and assessed correlations between BART scores, psychological metrics, and risk-taking behavior. Our correlation analysis shows that BART scores significantly correlated with sensation seeking and risky decision-making, and our group analysis on subsamples of participants with high and low BART scores demonstrates significant differences in sensation seeking, risky decision-making, and sex. Furthermore, risky decision-making during VR driving could significantly be predicted based on the low- or high-BART group allocation.

Since one of the main goals of the BART is to predict real-world risk-taking behavior [44], our VR BART offers an important advantage in that it provides participants with a more naturalistic environment. While previous studies used risky driving tasks in a driving simulator [45] or self-report questionnaires [46, 47] to find group differences between high- and low-risk-taking groups, our study extends those previous findings since our VR BART can also predict risky decision-making in an emergency situation. Additionally, it was found that average driving speed in an emergency situation significantly correlated with the BART scores, which implies that the BART score correlates with emergency decision-making and consistent risky behavior in an emergency situation.

Additionally, it was found that sensation-seeking significantly predicts BART scores. Although an earlier meta-analysis of correlations between sensation seeking and BART scores showed fluctuations in correlations [9], our study shows a significant correlation between BART scores and sensation seeking for a relevant sample size, which corroborates the notion that higher immersiveness on the BART may induce emotional arousal that contributes to finding significant correlations [9] between sensation-seeking and BART scores. However, while several earlier studies reported that sensation seeking correlated with risky driving behavior [9, 11, 21–24, 48], we did not find such correlations in our VR driving task. A potential reason for this might be that sensation-seeking is associated with consistent rather than momentary decision-making. This is supported by the results of our previous study [49], which showed significant correlations between average driving speed and sensation seeking but no correlations between emergency decision-making and driving speed.

However, no significant contributions of impulsivity or age to the BART scores were found. This may be because the distinction between emotion-related impulsivity and other impulsivity types. Emotion-related impulsivity occurs when people experience heightened emotional states [50], which may not be induced by the VR BART; thus, emotion-related impulsivity may not have affected participants’ decision to pump more or less in our study. As a result, impulsivity showed no significant correlation with the BART scores. Furthermore, a previous study showed age effects on BART scores in a comparison between young and older adults [51]. However, since our study sample was limited to younger adults, no significant age effects were found. Future studies must extend the participant age range to detect potential differences between younger and older adults on the VR BART.

This is the first study to test the potential of VR BART. However, there are several limitations warranting further research. First, this study had a no-control condition that measured BART without VR. A previous study showed inconsistent correlations between psychological metrics and desktop BART scores [9], and different survey methods like mobile phones also did not find significant correlations [52]. However, to validate the influences of VR, future studies must implement experimental conditions such as participants wearing a head-mounted display and performing tasks with 2D or the same experiment on a desktop. Second, because this study’s participants did not receive any further incentive for the pump, risky decision-making in BART may differ from real-world decision-making. However, influences of real and hypothetical incentives are controversial; several studies reported no differences between real and hypothetical incentives across different paradigms [53–55], while others reported that when the amount of real incentive is increased, decision-making and neural activity may differ considering hypothetical conditions [56–58]. Hence, future studies must test the influences of real incentives in VR BART, which may change the real incentive amount to investigate its effect on risky decision-making. Third, this study used median value-based artificial categorization; hence, results from the group split have potential limitations in generalization [43]. However, a previous study showed that artificial categorization might handle highly skewed data [59], and BART scores in this study are not normally distributed, making group split the preferred method. Fourth, we used logistic regression analysis for regression models to predict risky decision-making from psychological metrics based on the guidelines of the previous study [60]; however, our sample size may not be enough to develop such a model. Therefore, to validate the model that BART subgroup assignment with sex can predict risky decision-making, future studies must perform additional VR BART studies. Finally, this study only used one traffic scenario with limited psychological metrics to predict risky decision-making in driving. Future studies must implement multiple risky driving scenarios (e.g., rear-end and side collisions) with additional sensor data (e.g., eye-tracking, electrocardiogram, skin conductance, and electroencephalogram) to enhance prediction accuracy and predict various risky decision-making in real-world from VR BART and psychological metrics.

## Conclusion

The present study used a new VR BART to assess the relationships between BART scores, personality features, and risky decision-making during VR driving. Overall, the findings indicate that sensation seeking, risky decision-making, average driving speed during an emergency, and sex are associated with BART scores. Although there is still a gap between VR and real-world decision-making, our new VR BART showcases the potential to predict naturalistic decision-making. Previous studies have indicated that VR can enhance ecological validity [61, 62], and this study’s VR BART can enhance the validity and reliability of laboratory-based experiments. Future studies can add control conditions using head-mounted displays without 3D and provide incentives for enhancing ecological validity to ensure BART predicts better risky decision-making. Moreover, our VR BART can help inspire the implementation of more realistic behavioral tasks in other disciplines. These tasks will allow researchers to model and predict behavior to identify people who exhibit risky behavior (reckless driving, alcohol addiction, gambling) and provide proper treatment or counseling to prevent future problems.
